# Intrapartal cardiotocographic patterns and hypoxia-related perinatal outcomes in pregnancies complicated by gestational diabetes mellitus

**DOI:** 10.1007/s00592-021-01756-0

**Published:** 2021-06-21

**Authors:** Mikko Tarvonen, Petteri Hovi, Susanna Sainio, Piia Vuorela, Sture Andersson, Kari Teramo

**Affiliations:** 1 Department of Obstetrics and Gynecology, University of Helsinki, and Helsinki University Central Hospital, Helsinki, Finland; 2 National Institute for Health and Welfare (THL), Helsinki, Finland; 3 Finnish Red Cross Blood Transfusion Service, Helsinki, Finland; 4 Health and Social Welfare Department, Vantaa, Finland; 5 Pediatric Research Center, Children’s Hospital, University of Helsinki, and Helsinki University Central Hospital, Helsinki, Finland

**Keywords:** Birth cohort, Intrapartum cardiotocography, Fetal heart rate, Fetal asphyxia, Gestational diabetes mellitus, Perinatal outcome

## Abstract

**Aims:**

In previous reports, cardiotocographic (CTG) fetal heart rate (FHR) monitoring has shown only limited benefits in decreasing adverse perinatal outcomes in pregnancies complicated by gestational diabetes mellitus (GDM). The aim of the present study was to evaluate whether an association exists between the recently reported ZigZag pattern (FHR baseline amplitude changes of > 25 bpm with a duration of 2–30 min) and asphyxia-related neonatal outcomes in GDM pregnancies.

**Methods:**

Intrapartal CTGs were recorded in a one-year cohort of 5150 singleton childbirths. The following CTG changes were evaluated: ZigZag pattern, saltatory pattern, late decelerations, episodes of tachycardia and bradycardia, reduced variability, and uterine tachysystole. The cohort was divided into three groups: women with GDM, women with normal oral glucose tolerance test (OGTT), and women with no OGTT performed. Umbilical artery (UA) blood gases, Apgar scores, neonatal respiratory distress, and neonatal encephalopathy were used as outcome variables.

**Results:**

GDM was diagnosed in 624 (12.1%), OGTT was normal in 4115 (79.9%), and OGTT was not performed in 411 (8.0%) women. Hypoxia-related ZigZag patterns (OR 1.94, 95% CI 1.64–2.34) and late decelerations (OR 1.65, 95% CI 1.27–2.13) of FHR, as well as a greater risk of fetal asphyxia (UA pH < 7.10 and/or UA BE < -12.0 meq/L and/or Apgar scores < 7 at 5-min) (OR 6.64, 95% CI 1.84–12.03) were observed in those with GDM compared with those without GDM.

**Conclusions:**

GDM is associated with intrapartal ZigZag pattern and late decelerations, cord blood acidemia and low 5-min Apgar scores at birth indicating increased occurrence of fetal hypoxia in GDM pregnancies.

## Introduction

Gestational diabetes mellitus (GDM) is the most common medical disorder in pregnancy [1]. It is estimated that about 20 million or 16% of live childbirths worldwide in 2019 were associated with some form of maternal hyperglycemia in pregnancy, of which 84% were due to GDM [2]. GDM is increasing globally mainly as a result of increasing overweight and obesity in women of childbearing age [3]. In Finland, GDM was diagnosed in 19% of all childbirths in 2019 and in 25% of childbirths with maternal age 35 years or older [4].

A linear relationship has been found between fasting, 1-h and 2-h glucose values of the OGTT and perinatal complications [5]. Furthermore, an adequate treatment of GDM can lower the risk of perinatal complications to about the same level as for pregnancies without GDM [3, 5–7].

During labor, uterine contractions result in a reduction in uteroplacental perfusion, causing transient fetal and placental hypoxia. A healthy term fetus with a normally developed and functioning placenta is able to adapt to this transient hypoxia without adverse consequences. Women with GDM have higher mean fasting and 2-h post-prandial glucose values compared with mothers without GDM, which may lead to an increase in fetal blood glucose concentration and further adversely affect fetal oxygenation [8].

Cardiotocographic (CTG) monitoring of fetal heart rate (FHR) aims to predict and diagnose fetal hypoxia before fetal compromise occurs [9]. However, previous studies have shown only limited benefit of CTG monitoring in decreasing the risk of adverse fetal and neonatal outcome in GDM pregnancies [10]. Recently, a novel and more detailed analyses of FHR patterns have shown that recognition of the ZigZag pattern (baseline amplitude changes of > 25 bpm with a duration of 2–30 min) may improve the CTG as a screening tool of intrapartum fetal hypoxia in term pregnancies [11–13]. The ZigZag pattern precedes late decelerations in the majority of the cases.^11^ However, it is not known whether the ZigZag pattern of FHR is a sign of imminent fetal compromise in pregnancies complicated by GDM. We hypothesize that GDM may contribute to intrapartum fetal hypoxia and this process can be identified early by the ZigZag pattern in CTG recording during these childbirths.

The aim of the present study was to evaluate whether an association exists between ZigZag pattern of FHR during the last two hours of labor and asphyxia-related neonatal outcomes in GDM pregnancies.

## Material and methods

### Study population

**Fig. 1:**
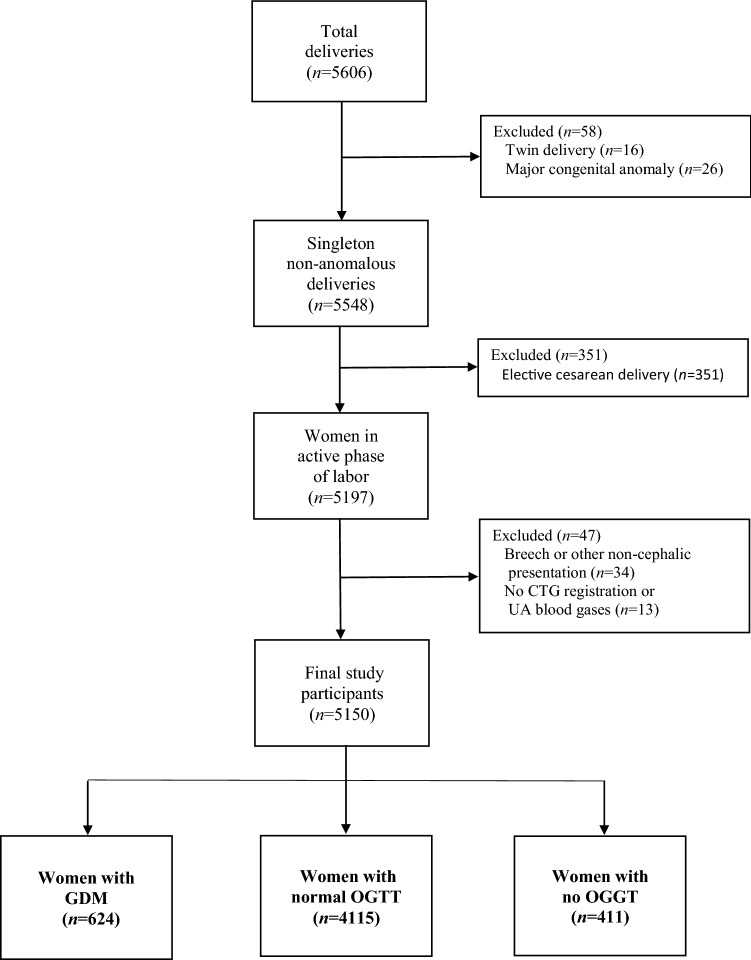
Flow chart of the study participants and grouping of the parturients according to the GDM present or absent or with no OGTT performed. *CTG*, Cardiotocography: *OGTT*, Oral glucose tolerance test

The cohort consisted of 5150 singleton childbirths with continuously monitored CTG tracings during the last two hours of labor with ≥ 33 weeks of gestation at the Maternity Hospital, Helsinki University Hospital (HUS) between January 1 and December 31, 2012. In the Helsinki metropolitan area, the Maternity Hospital took care of low-risk childbirths, excluding, for example, preterm births < 33 weeks of gestation, childbirths of women with severe preeclampsia or type 1 diabetes, and fetuses with severe intrauterine growth restriction. The one-year obstetric cohort is the same as in our recent report on term deliveries, except for the inclusion of preterm (from 33.0 to 36.9 weeks of gestation) deliveries in the present study [11]. All women in the cohort were in the active phase of labor with regular uterine contractions. Preterm pregnancies with < 33 weeks of gestation, non-cephalic presentations, elective cesarean delivery without labor contractions, major congenital malformations, and cases with missing CTG registration or missing UA blood gas results were excluded from the study cohort (Fig. 1). FHR was recorded via a scalp electrode in 91.1% of the cases.

According to the OGTT, the cohort was divided into three groups: GDM, normal OGTT, and no OGTT performed (Fig. 1). The maternal and delivery-related characteristics of the three groups are presented in Table 1.

**Table 1:** Maternal and delivery-related characteristics when GDM is present or absent, or without OGTT performed

Maternal variables	Total	GDM	OGTT normal	No OGTT performed	*P* value for GDM vs OGTT normal	*P* value for GDM vs no OGTT performed	*P* value for OGTT normal vs no OGTT performed
Number	5150	624 (12.1)	4115 (79.9)	411 (8.0)			
Maternal age ≥ 35 years	1276 (24.8)	177 (28.4)	998 (24.3)	101 (24.6)	0.017*	0.25	0.89
Obesity, prepregnancy BMI ≥ 30.0 (kg/m^2^)	565 (11.0)	120 (19.2)	444 (10.8)	0 (0.0)	< 0.001*	< 0.001*	< 0.001*
Nulliparous	2855 (55.4)	347 (55.6)	2273 (55.2)	235 (57.2)	0.73	0.62	0.45
Parous	2295 (44.6)	277 (44.4)	1842 (44.8)	176 (42.8)			
Gestational age at delivery (wk)	40.1 (± 1.3)	40.0 (± 1.1)	40.1 (± 1.3)	40.2 (± 1.4)	< 0.001†	< 0.001†	< 0.001†
Smoking	471 (9.1)	58 (9.3)	381 (9.3)	32 (7.7)	0.84	0.29	0.13
Previous macrosomia	113 (2.2)	19 (3.0)	92 (2.2)	2 (0.5)	0.41	0.004*	0.24
Immediate family history of type 2 diabetes	667 (13.0)	121 (19.4)	541 (13.1)	4 (1.0)	< 0.001*	< 0.001*	< 0.001†
Previous gestational diabetes	291 (5.7)	89 (14.3)	202 (4.9)	0 (0.0)	< 0.001*	< 0.001*	< 0.001†
Present gestational diabetes							
Diet-treated	571 (11.1)	571 (91.5)	0 (0.0)	0 (0.0)	NA	NA	NA
Metformin-treated	44 (0.9)	44 (7.1)	0 (0.0)	0 (0.0)	NA	NA	NA
Insulin-treated	9 (0.2)	9 (1.4)	0 (0.0)	0 (0.0)	NA	NA	NA
Preeclampsia	151 (2.9)	19 (3.2)	132 (3.1)	5 (1.2)	0.94	0.39	0.66
Maternal fever at delivery ≥ 38.0 °C	128 (2.5)	20 (3.2)	97 (2.4)	11 (2.7)	0.37	0.59	0.87
Type of onset of labor							
Spontaneous	3975 (77.2)	462 (74.0)	3200 (77.8)	313 (76.2)	0.020*	0.40	0.46
Induction	1175 (22.8)	162 (26.0)	915 (22.2)	98 (23.8)			
Oxytocin augmentation	2893 (56.2)	368 (59.0)	2295 (55.8)	230 (56.0)	0.07	0.26	0.91
Duration of first stage of labor (min)	501 (± 234)	516 (± 238)	498 (± 233)	500 (± 241)	0.08	0.29	0.87
Duration of second stage of labor (min)	53 (± 40)	55 (± 42)	52 (± 40)	54 (± 40)	0.10	0.70	0.33
Mode of delivery							
Spontaneous vaginal	4121 (80.0)	463 (74.2)	3334 (81.0)	324 (78.8)	< 0.001†	0.09	0.28
Vacuum extraction	521 (10.1)	64 (11.9)	408 (9.9)	49 (11.9)	0.16	0.92	0.20
Cesarean (elective excluded)	508 (9.9)	88 (14.1)	382 (9.3)	38 (9.2)	0.003*	0.022*	0.93

Data are mean ± SD or number (%). * significant when present. † significant when absent

*GDM* Gestational diabetes mellitus, *OGTT* Oral glucose tolerance test, *BMI* Body mass index, *NA* Not applicable due to selection criteria

All women were screened according to the Finnish National Current Care guidelines for gestational diabetes mellitus [4, 14]. The guidelines recommend that all women should be screened for GDM, excluding those with a very low risk (a primipara ≤ 25 years old with a body mass index (BMI) < 25 kg/m2, and without a family history of type 2 diabetes, or a parous woman < 40 years old with a BMI < 25 kg/m2, without a family history of type 2 diabetes and without previous GDM or fetal macrosomia, i.e., birth weight z-score > 2.0 SD-units) [14]. A 2-h OGTT for screening of GDM with 75 g glucose was performed at 24–28 weeks of gestation with the following cut-off values: fasting ≥ 5.3 mmol/l, 1 h value ≥ 10.0 mmol/l, and 2 h value ≥ 8.6 mmol/l. A single abnormal value was diagnostic for GDM [14].

### Data sources

The CTGs were recorded using Avalon® FM40 and FM50 (Philips Healthcare, Andover, MA, USA) fetal monitors. All CTG recordings were stored in visual and electronic forms in the Milou® (Medexa, Limhamn, Sweden) CTG database at the Data Analysis and Management Department of HUS. After delivery, CTG recordings were coded and printed on paper for two experienced perinatologists for interpretation. The clinical data were collected from electronic obstetric patient records (Obstetrix®, Obstetrix Medical Group, Englewood, CO, USA). The results of the oral glucose tolerance test (OGTT) were obtained from the HUS Weblab Clinical® laboratory information system.

### Evaluation of intrapartum CTG recordings

Two experienced perinatologists (S.S. and K.T.) evaluated the CTG recordings independently and without knowing the maternal, fetal or neonatal data, and perinatal outcomes in order to assess the following CTG changes: ZigZag pattern, saltatory pattern, late decelerations, episodes of bradycardia and tachycardia, reduced FHR variability, and uterine tachysystole. Only concordant CTG changes between the evaluators were used in the analyses. The findings were classified according to the FIGO (The International Federation of Gynecology and Obstetrics) guidelines on intrapartum fetal monitoring with the exception the ZigZag pattern (see below) [15].

**Fig. 2:**
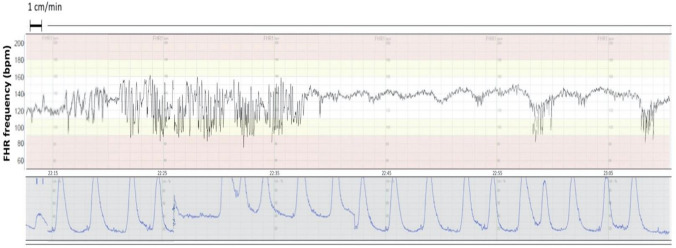
Intrapartum CTG recording at 41 + 1 weeks of pregnancy of a 36-year-old nullipara diagnosed with diet-treated GDM. At left, normal baseline FHR frequency (120 bpm/min) and normal variability followed by ZigZag pattern. A 17-min ZigZag episode is followed by repetitive late decelerations. The first stage of labor, cervix dilated 8 cm. No oxytocin augmentation was used. During the ZigZag pattern, no uterine hypertonus was observed, but the change of maternal position and movement of the abdominal toco transducer. A macrosomic male fetus, birth weight 4502 g, birth weight z-score + 2.1 SD, was born vaginally spontaneously 110 min after the occurrence of ZigZag pattern. Umbilical cord blood gas analysis showed acidemia: UA pH 7.05, UA BE -12.4 mmol/L, UA pO_2_ 1.7 kPa. Apgar scores of 6 and 8 at 1 and 5 min, respectively. FHR was recorded via scalp electrode with paper speed 1 cm/min

Normal baseline FHR was defined as a baseline between 110 and 160 bpm. Normal FHR variability was defined as baseline amplitude changes of 5–25 bpm. The ZigZag FHR pattern was defined as FHR baseline amplitude changes of > 25 bpm with a duration of 2–30 min [11]. The definition of the ZigZag pattern differs from the saltatory pattern in its duration and uniformity of the trace. The saltatory pattern was defined as FHR baseline amplitude changes of > 25 bpm and a duration of > 30 min. Late decelerations were defined as U-shaped decreases of FHR of > 15 bpm occurring late in relation to uterine contractions. In the presence of a tracing without accelerations and with reduced variability, the definition of late decelerations included also those with an amplitude of 10–15 bpm. Tachycardia was defined as a baseline frequency above 160 bpm lasting for more than 10 min. According to the FIGO guideline, FHR values between 100 and 110 bpm may occur in normal fetuses, especially in postdate pregnancies. Thus, in the present study, a bradycardia episode was defined as a baseline frequency below 100 bpm lasting for more than 3 min. The reduced variability was defined as an amplitude below 5 bpm for more than 10 min and that of uterine tachysystole as the occurrence of more than 5 contractions during a 10 min period. Figure 2 shows a CTG recording with ZigZag pattern followed by late decelerations.

### Maternal and delivery-related variables

The following maternal and delivery-related variables were determined: maternal age, prepregnancy BMI, ethnicity, mode of delivery, type of onset of labor (spontaneous or induction), oxytocin augmentation, gestational age, family history of type 2 diabetes, parity, present or previous GDM, previous fetal macrosomia, preeclampsia, maternal fever ≥ 38.0 °C, and smoking.

### Fetal and neonatal variables

The following fetal and neonatal variables were determined: gestational age at delivery, fetal sex, birth weight z-score, umbilical artery (UA) pH, base excess (BE), and pO_2_, Apgar scores at 1 and 5 min, need for intubation and resuscitation, neonatal intensive care unit (NICU) admission, and neonatal encephalopathy. According to the hospital`s general practice, UA blood was routinely collected from a double-clamped cord for pH and blood gas analyses in all childbirths. Fetal asphyxia was defined as UA pH < 7.10 and/or UA BE < -12.0 meq/L and/or 5-min Apgar scores < 7 [16, 17]. Neonatal respiratory distress was defined as the need for continuous positive airway pressure (CPAP) delivered using a T-piece-based infant resuscitator Neopuff® (Fisher & Paykel Healthcare Limited, Auckland, New Zealand) and/or intubation.

### Data analyses

We analyzed continuous variables by Analysis of variance (ANOVA), Kruskal–Wallis test and Mann–Whitney U-test. Pearson`s Chi-square and Fisher's Exact Probability test were used for categorical variables. All tests were two-sided. Values of *P* < 0.05 were considered statistically significant.

Logistic regression analysis was used to evaluate whether GDM was associated with hypoxia-related CTG changes when the models included parity, type of onset of labor (spontaneous or induction), oxytocin augmentation, obesity (prepregnancy BMI ≥ 30.0 kg/m^2^), gestational age at delivery (preterm < 37 weeks, term, or postterm ≥ 42 weeks of gestation), maternal age ≥ 35 years, preeclampsia, maternal fever ≥ 38.0 °C, smoking, fetal sex, and fetal macrosomia (birthweight z-score > 2.0 SD-units). The logistic regression analysis was performed by R version 3.6.0 and the odds ratios (OR) and 95% confidence intervals (CI) were estimated by fitting logistic regression models.

## Results

### Prevalence and interpretation concordance of CTG change

The prevalences of CTG features in women with or without GDM, and in women in whom OGTT was not performed, are presented in Table 2. Of the 5150 childbirths, only CTG changes that were concordant between the two perinatologists were used in the study. Therefore, from the total of 20 562 CTG changes identified of the 5150 CTG recordings, 17 765 concordant CTG changes were used in the final analyses. For the separate CTG changes, the concordance was as follows: for ZigZag pattern 87.2%, for late decelerations 82.1%, for bradycardia episodes 94.0%, for tachycardia episodes 92.1%, for reduced variability 78.3%, and for uterine tachysystole 85.4%. The overall concordance of CTG patterns between the two perinatologists was 86.3%.

**Table 2:** Neonatal characteristics and CTG features when GDM is present or absent, or without OGTT performed

Neonatal variables	Total	GDM	OGTT normal	No OGTT performed	*P* value for GDM vs OGTT normal	*P* value for GDM vs no OGTT performed	*P* value for OGTT normal vs no OGTT performed
Number	5150	624 (12.1)	4115 (79.9)	411 (8.0)			
Neonate sex							
Female	2502 (48.6)	249 (39.9)	2053 (49.9)	200 (48.7)	< 0.001*	0.005*	0.45
Male	2648 (51.4)	375 (60.1)	2062 (50.1)	211 (51.3)			
Macrosomia (Birth weight z-score > 2.0 SD-units)	151 (2.9)	51 (8.2)	91 (2.0)	9 (2.2)	0.001*	0.030*	0.80
Preterm (< 37.0 wk)	162 (3.1)	15 (2.4)	134 (3.3)	13 (3.2)	0.06	0.27	0.79
Postterm (≥ 42.0 wk)	482 (9.4)	21 (3.4)	420 (10.2)	41 (10.0)	< 0.001†	< 0.001†	0.74
CTG features							
ZigZag pattern	582 (11.3)	97 (15.5)	446 (10.8)	39 (9.5)	< 0.001*	0.006*	0.53
Saltatory pattern	0 (0.0)	0 (0.0)	0 (0.0)	0 (0.0)	1.00	1.00	1.00
Late decelerations	2100 (40.8)	331 (53.0)	1599 (38.9)	170 (41.4)	< 0.001*	< 0.001*	0.28
Bradycardia episodes	2724 (52.9)	282 (45.2)	2210 (53.7)	232 (56.4)	< 0.001†	< 0.001†	0.21
Reduced variability	1892 (36.7)	240 (38.5)	1505 (36.6)	147 (35.8)	0.36	0.22	0.70
Tachycardia episodes	721 (14.0)	146 (23.4)	529 (12.9)	46 (11.2)	< 0.001*	< 0.001*	0.41
Uterine tachysystole	228 (4.4)	56 (9.0)	160 (3.9)	12 (2.9)	< 0.001*	< 0.001*	0.63
No CTG changes	1155 (22.4)	91 (14.6)	977 (23.7)	87 (21.2)	< 0.001†	0.010†	0.23
1 min Apgar score < 7	72 (1.4)	25 (4.0)	42 (1.0)	5 (1.2)	< 0.001*	< 0.001*	0.75
5 min Apgar score < 7	12 (0.2)	8 (1.3)	4 (0.1)	0 (0.0)	< 0.001*	0.041*	0.09
UA pH	7.27 (± 0.08)	7.24 (± 0.09)	7.27 (± 0.08)	7.27 (± 0.09)	< 0.001*	< 0.001*	0.21
UA BE (meq/L)	− 3.8 (± 6.6)	− 4.9 (± 6.4)	− 3.7 (± 6.7)	− 3.6 (± 6.6)	< 0.001*	< 0.001*	0.10
UA pO_2_ (kPa)	3.2 (± 1.0)	3.1 (± 0.9)	3.2 (± 1.0)	3.2 (± 1.0)	0.015*	0.002*	0.33
UA acidemia							
UA pH < 7.10	150 (2.9)	33 (5.3)	106 (2.6)	11 (2.7)	0.001*	0.001*	0.69
UA BE < -12.0 (meq/L)	40 (0.8)	18 (2.9)	20 (0.5)	2 (0.5)	0.003*	0.028*	0.85
Intubation for resuscitation	21 (0.4)	10 (1.6)	11 (0.3)	0 (0.0)	0.001*	0.021*	0.77
NICU admission for respiratory distress	208 (4.8)	60 (9.6)	127 (3.1)	21 (5.1)	< 0.001*	< 0.001*	0.06
Neonatal encephalopathy	2 (0.04)	0 (0.0)	2 (0.04)	0 (0.0)	0.23	1.00	0.40

Data are mean ± SD or number (%). * significant when present. † significant when absent

*GDM* Gestational diabetes mellitus, OGTT Oral glucose tolerance test, *BE* Base excess, *NICU* Neonatal intensive care unit, *UA* Umbilical artery

### Hypoxia-related CTG changes

Fetuses with a ZigZag pattern had a higher risk of intrapartum fetal asphyxia (UA pH < 7.10 and/or UA BE < -12.0 meq/L and/or 5-min Apgar scores < 7) compared with cases without the pattern among women with GDM (OR 1.90, 95% CI 1.31–2.72). The finding was similar for those with late decelerations (OR 1.55, 95% CI 1.19–1.91).

Furthermore, fetuses of GDM women were more likely to have ZigZag pattern (OR 1.94, 95% CI 1.64–2.34) and late decelerations (OR 1.65, 95% CI 1.27–2.13) than fetuses of women with no GDM or in women with no OGTT. Logistic regression analysis revealed that adjustment for maternal and fetal risk factors attenuated the association between GDM and hypoxia-related FHR patterns only marginally (Table 3).

**Table 3:** Primary asphyxia-related outcomes of fetuses of women with GDM as compared with fetuses of women without GDM

Fetal and neonatal asphyxia-related outcomes	GDM vs. no-GDM	
	Crude OR (95% CI)	Adjusted* OR (95% CI)
Hypoxia-related FHR patterns		
ZigZag pattern	1.94 (1.64–2.34)	1.59 (1.31–2.12)
Late decelerations	1.65 (1.27–2.13)	1.50 (1.17–1.97)
Fetal distress		
UA pH < 7.10	5.82 (2.30–17.14)	5.74 (2.24–17.05)
UA pH < -12.0 meq/L	12.03 (2.87–55.16)	11.42 (2.23–54.39)
5-min Apgar score < 7	4.71 (2.60–29.32)	4.75 (1.64–29.36)
Fetal asphyxia^	6.64 (1.84–12.03)	6.19 (1.46–11.60)
CPAP	3.19 (2.60–3.91)	3.23 (2.65–3.98)
Intubation	10.02 (3.31–28.00)	9.96 (3.27–27.93)
Neonatal respiratory distress †	3.61 (2.56–5.05)	3.67 (2.52–5.12)

^*^ Adjusted for parity, induction of labor obesity (prepregnancy BMI ≥ 30.0 kg/m^2^), oxytocin augmentation, gestational age at delivery (preterm < 37 weeks and postterm ≥ 42 weeks of gestation), maternal age ≥ 35 years, preeclampsia, maternal fever ≥ 38.0 °C, smoking, fetal sex, and fetal macrosomia (birthweight z-score > 2.0 SD-units)

^ UA pH < 7.10 and/or UA BE < -12.0 meq/L and/or 5-min Apgar scores < 7

^†^ Need for CPAP and/or intubation

*CI* Confidence interval, *CPAP* Continuous positive airway pressure, *FHR* Fetal heart rate, GDM Gestational diabetes mellitus, *OR* Odds ratio, *UA* Umbilical artery

The ZigZag pattern occurred in term pregnancies (≥ 37 weeks of gestation) only. The ZigZag pattern occurred in 304 of the 582 cases (52.2%) during the first stage of labor (cervix dilated < 10 cm) and in 278 cases (47.8%) during the second stage (cervix dilated 10 cm) (*P* = 0.17 for difference). No differences were observed in the duration of the first and the second stages of labor between the three study groups (Table 1). In the total study population, ZigZag pattern occurred in 91.2% (531/582) of the CTG recordings together with late decelerations. ZigZag pattern preceded late decelerations in 88.7% (471/531) of the cases. A normal FHR preceded the ZigZag pattern in 90.4% (526/582) of the cases, whereas after ZigZag episodes, a normal FHR pattern was observed in 0.9% (5/582) only.

Fetuses of GDM mothers with two abnormal OGTT values had the strongest association with the intrapartal ZigZag pattern (OR 2.27, 95% CI 1.82–2.85). Fasting (OR 1.81, 95% CI 1.36–2.39) hyperglycemia or an abnormal 2-h (OR 1.60, 95% CI 1.19–2.14) OGTT value correlated with the occurrence of ZigZag pattern, whereas no association was found between this FHR pattern and an abnormal 1 h (OR 0.91, CI 95% 0.70–1.17) OGTT value.

### Other CTG changes

In all three study groups, bradycardia episodes occurred in approximately half of the cases (Table 2). The majority (78.0%) of the bradycardia episodes appeared during the last 30 min before birth. The presence of episodes with reduced baseline variability was relatively common in all the three study groups (Table 2). The vast majority (92.6%) of the episodes of reduced variability were preceded and followed by normal baseline variability and FHR accelerations.

Furthermore, fetuses of women with GDM had more often tachycardia episodes than women without GDM (OR 1.73, 95% CI 1.39–2.13) (Table 2). However, the vast majority (83.4%) of the tachycardia episodes were preceded and followed by normal frequency of baseline FHR, normal baseline variability, and FHR accelerations. No significant association was found between bradycardia episodes, or reduced variability and GDM (Table 2).

Fetal macrosomia occurred 4.3 times more often in GDM pregnancies compared with pregnancies without GDM (OR 4.31, 95% CI 2.94–6.26). Of the CTG changes, fetal macrosomia was associated with uterine tachysystole (OR 3.13 95% CI 1.84–5.11), but not with ZigZag pattern, late decelerations or episodes of tachycardia. The prevalence of cases with uterine tachysystole was increased in GDM women regardless of the fact that no difference was observed in the use of oxytocin between the three study groups (Table 2).

### Fetal and neonatal outcome variables associated with GDM

Table 3 presents the primary fetal and neonatal asphyxia-related outcomes in women with GDM as compared with women without GDM. Newborn infants of mothers with GDM had 6.6-fold risk of intrapartum fetal asphyxia (UA pH < 7.10 and/or UA BE < -12.0 meq/L and/or 5-min Apgar scores < 7) compared with women with no GDM or no OGTT (Table 3). No differences in the occurrence of cord blood acidemia at birth or low 1- or 5 min Apgar scores were found between the cases with normal OGTT and no OGTT performed (Table 2). In women with GDM, newborn infants had 3.6-fold risk of respiratory distress compared with those with normal OGTT and no OGTT performed (Table 3).

## Discussion

### Principal findings

The present study has three important findings. Firstly, the hypoxia-related ZigZag pattern and late decelerations are significantly more common in GDM pregnancies compared with those without GDM. Secondly, during the last two hours of labor, the occurrence of ZigZag pattern in CTG tracings correlates with intrapartum fetal asphyxia among fetuses of mothers with GDM. Thirdly, GDM is associated with clinical signs of perinatal hypoxia, UA acidemia, low Apgar scores at birth, neonatal respiratory distress, and need for intubation.

### Interpretation

The underlying pathophysiology of GDM resulting in fetal hypoxia has been a matter of debate. In GDM pregnancies, mainly placental functional changes occur [18, 19]. However, if impaired glucose metabolism is diagnosed in early pregnancy as a result of more severe form of GDM, structural placental changes have been observed [20, 21]. Furthermore, GDM elicits major changes in the expression of placental genes with a prominent increase in markers and mediators of inflammation [18, 19, 22]. Mild constant hyperglycemia combined with short periods of postprandial hyperglycemia has been shown to increase fetal insulin production.[18] In sheep fetuses, chronic hyperglycemia and secondary hyperinsulinemia increase oxygen consumption and reduce blood oxygen levels [23, 24]. When fetal blood oxygen levels decrease as a result of hyperglycemia, fetal plasma erythropoietin (EPO) levels increase sharply due to increasing fetal EPO synthesis [25]. Consequently, both placental abnormalities and increased oxygen consumption may lead to intrauterine fetal hypoxia [8, 25]. Although the pathophysiology behind fetal hypoxia in hyperglycemic pregnancies has been examined mainly in animal studies and in type 1 diabetes in humans, [23–25] elevated markers of fetal hypoxia, such as abnormal cord blood acid–base status and increased cord EPO levels at birth, occur also in GDM pregnancies, [25, 26] suggesting the same underlying pathogenesis. Consequently, throughout the journey of labor, the fetus of a diabetic mother may undergo increased hypoxic stress and therefore, abnormal FHR changes will be seen on the CTG tracing, such as increase in FHR variability, i.e., ZigZag pattern.

In our recent reports, the ZigZag pattern during the last 2 h of labor is associated with fetal intrapartum hypoxia, as indicated by high cord blood EPO levels, cord blood acidemia, and severe neonatal complications [11, 27]. The ZigZag pattern typically precedes late decelerations in CTG tracings, and the fact that normal FHR pattern precedes the ZigZag pattern in the vast majority of the cases, indicates that the ZigZag pattern is an early sign of fetal hypoxia [11, 27]. Furthermore, the ZigZag pattern is independently associated with intrapartum fetal asphyxia even when the pattern occures in the absence of late decelerations [11]. According to our present findings, the ZigZag pattern appears to be an adequate and early indicator of fetal hypoxia also in GDM pregnancies. The increased occurrence of the ZigZag pattern followed by late decelerations in intrapartum CTG tracings is a new observation in GDM pregnancies.

In a 1995 study by Kjos et al., CTG recording was performed on 2134 women with diabetes (1388 women with GDM) [28]. The factors most predictive of cesarean delivery for fetal distress were the presence of late decelerations, nonreactive FHR, and the combination of both nonreactive FHR and FHR decelerations [28]. In a 2011 study by Yli et al., intrapartum CTG monitoring was performed on 413 women with diabetes (307 women with GDM), among whom a preterminal FHR pattern (i.e., FHR pattern with absent FHR baseline variability with or without bradycardia) was more common in fetuses of mothers with type 1 diabetes or GDM than in pregnancies of nondiabetic mothers [29]. However, in the present study, no association was found between episodes of reduced FHR baseline variability or bradycardia and GDM.

Both in pregnancies with and without GDM, bradycardia episodes occurred in approximately 50% of the fetuses, the majority of which took place in the active second stage of labor. This suggests that the bradycardia episodes were caused by fetal head compression, [30] baroreflex activation following increased blood pressure during an overstretched or compressed umbilical cord, [31] or the peripheral chemoreflex triggered by transient periods of acute fall in oxygen tension mediated by frequent uterine contractions [32].

Our observations are in agreement with previous reports that show that women with GDM have a higher risk of low 1- and 5-min Apgar scores [33] and of nonelective operative delivery [34, 35] than women with normal OGTT. Furthermore, in the present and previous studies, GDM was associated with increased risk of neonatal respiratory distress also in term (gestational age ≥ 37 weeks) pregnancies [35, 36]. Furthermore, we found an association between maternal GDM and the occurrence of fetal cord blood acidemia at birth. A similar relation in women with type 1 diabetes or insulin-treated GDM has been observed, [37, 38] but not in women with mild (diet-treated) GDM [34, 35, 39]. Accordingly, several studies conclude that no routine fetal assessment by CTG is needed in diet-controlled term GDM pregnancies without secondary complications [10, 40–42].

### Strengths and limitations

Strengths of this study are the large number of the study subjects, the use of well-defined criteria of different FHR changes, the high number and quality of the scalp-monitored CTG recordings, and the high concordance between the two experts evaluating the CTG recordings. This is distinctly higher, both in interpretation of a single CTG change as well as in the overall concordance than in previously reported studies [43–45]. Although OGTT was not performed in 8.0% of women in the cohort, we find it unlikely that there would be many women with undiagnosed GDM among this group. There was no difference in fetal and neonatal outcome characteristics between women with no GDM and those with no OGTT performed. We, therefore, conclude that very few of the 411 women in whom OGTT was not done, had GDM. Moreover, none of the women were obese (BMI ≥ 30.0 kg/m^2^) of those with no OGTT performed.

Despite its robust size, our study has some limitations. The first of which is the retrospective study design. Secondly, the evaluation of the CTG recordings was restricted to the last 2 h prior to delivery. Thirdly, fetal macrosomia occurred four times more often in pregnancies of GDM mothers. The absence of data regarding antenatal glycemic controls may contribute to explain fetal hypoxia in macrosomic newborn infants. Finally, it may be difficult to extrapolate our results to the general obstetric population because our study was conducted at a single university center. Therefore, a large population-based multicenter cohort study is needed to confirm these results in the general population.

## Conclusions

In conclusion, our findings confirm that maternal GDM may expose the fetus to increased risk of intrapartum hypoxia, which can be detected in CTG recordings as increased ZigZag FHR pattern and other hypoxia-related FHR changes and as asphyxia-related fetal and neonatal outcomes at birth. Understanding this process may improve the clinical decision making on intrapartum CTG recordings in GDM pregnancies and would enable the clinician to plan for possible interventions during labor. We suggest, based on timely recognition of ZigZag and repetitive late decelerations FHR patterns, that continuous CTG recording should be used routinely during labor of GDM women. In order to evaluate the potential long-term effects, future research should include follow-up of offspring with hypoxia-related changes, especially with the ZigZag pattern, in FHR recording during labor in pregnancies of GDM women.

## Data Availability

The data of this study are available on request from the corresponding author (MT). The data are not publicly available due to privacy and ethical restrictions.

## Abbreviations

BE: Base excess
BMI: Body mass index
CI: Confidence interval
CTG: Cardiotocograph
FHR: Fetal heart rate
GDM: Gestational diabetes mellitus
NICU: Neonatal intensive care unit
OGTT: Oral glucose tolerance test
OR: Odds ratio
UA: Umbilical artery
